# Constructing the ceRNA Regulatory Network and Combining Immune Cells to Evaluate Prognosis of Colon Cancer Patients

**DOI:** 10.3389/fcell.2021.686844

**Published:** 2021-10-07

**Authors:** Jiasheng Xu, Tianyi Ling, Siqi Dai, Shuwen Han, Kefeng Ding

**Affiliations:** ^1^Department of Colorectal Surgery and Oncology, Key Laboratory of Cancer Prevention and Intervention, Ministry of Education, The Second Affiliated Hospital, Zhejiang University School of Medicine, Hangzhou, China; ^2^Cancer Center, Zhejiang University, Hangzhou, China

**Keywords:** ceRNA regulatory network, immune cells, prognosis, evaluation model, colon cancer

## Abstract

**Objective:** This study was conducted in order to construct a competitive endogenous RNA (ceRNA) network to screen RNA that plays an important role in colon cancer and to construct a model to predict the prognosis of patients.

**Methods:** The gene expression data of colon cancer were downloaded from the TCGA database. The difference was analyzed by the R software and the ceRNA network was constructed. The survival-related RNA was screened out by combining with clinical information, and the prognosis model was established by lasso regression. CIBERSORT was used to analyze the infiltration of immune cells in colon cancer, and the differential expression of immune cells related to survival was screened out by combining clinical information. The correlation between RNA and immune cells was analyzed by lasso regression. PCR was used to verify the expression of seven RNAs in colon cancer patients with different prognoses.

**Results:** Two hundred and fifteen lncRNAs, 357 miRNAs, and 2,955 mRNAs were differentially expressed in colon cancer. The constructed ceRNA network contains 18 lncRNAs, 42 miRNAs, and 168 mRNAs, of which 18 RNAs are significantly related to survival. Through lasso analysis, we selected seven optimal RNA construction models. The AUC value of the model was greater than 0.7, and there was a significant difference in the survival rate between the high- and low-risk groups. Two kinds of immune cells related to the prognosis of patients were screened out. The results showed that the expression of seven RNA markers in colon cancer patients with different prognoses was basically consistent with the model analysis.

**Conclusion:** We have established the regulatory network of ceRNA in colon cancer, screened out seven core RNAs and two kinds of immune cells, and constructed a comprehensive prognosis model of colon cancer patients.

## Introduction

Colon cancer is a common tumor with high incidence in the world and ranks third in terms of mortality rate (Sung et al., 2005; Siegel et al., 2017). Colon cancer originates from intestinal epithelial cells in the colon. Its pathogenesis is influenced by many factors such as heredity (family history), environment, microorganism, living habits (excessive drinking, lack of exercise, high-fat and high-sugar diet), and inflammatory bowel disease (Johns and Houlston, 2001; Meyerhardt et al., 2003; Huxley et al., 2009; Terzić et al., 2010; Watson and Collins, 2011). Colorectal cancer is characterized by rapid metastasis and difficult treatment. Therefore, it is one of the diseases causing huge social and medical burden (Huxley et al., 2009; Watson and Collins, 2011). At present, the 5-year survival rate of regional and local colon cancer treated by various tests and surgical operations has been as low as 71% and 90%. However, for colon cancer with metastasis, the 5-year survival rate is only 14% (American Cancer Society Cancer Facts, 2019). More importantly, 25% of colon cancer patients have metastasis during diagnosis, and about 50% of colon cancer patients will eventually metastasize (Vatandoust et al., 2015). The causes of these serious consequences may be related to the effectiveness of the current treatment scheme. The current standard cancer treatment is surgical resection, radiotherapy, and chemotherapy (O’eilly et al., 1997). Therefore, it is very important to find a more effective treatment.

Long intergenic non-coding RNAs (lincRNAs) are non-coding RNAs with a length of more than 200 nucleotides (FANTOM Consortium et al., 2002; Guttman and Rinn, 2012). Most lincRNAs are located in the nucleus and only 15% are in the cytoplasm (Kapranov et al., 2007). As a competitive endogenous RNA (ceRNA), long non-coding RNA (lncRNA) can reduce the activity of microRNA (miRNA) and upregulate the expression of downstream genes regulated by miRNA through chelation (Tay et al., 2014). Studies have shown that ceRNA plays an important role in colon cancer, rectal cancer, glioma, bladder urothelial carcinoma, and other diseases (Qi et al., 2015; Xu et al., 2015). For example, lncRNA malat1 can induce the development of colon cancer by regulating the mir-129-5p/HMGB1 axis (Wu et al., 2018). At present, there are not many studies on the analysis of colon cancer by the ceRNA regulatory network. Therefore, it is of great significance to predict the prognosis of patients with colon cancer from the direction of ceRNA.

In this study, we screened out lncRNA, miRNA, and mRNA differentially expressed in colon cancer by analyzing a public database. Combined with several prediction databases, we constructed the key ceRNA network in colon cancer, and combined with clinical information, screened out seven RNAs closely related to the survival of patients with colon cancer. These seven RNAs can predict the prognosis of patients with colon cancer. Our results provide a promising therapeutic target and novel insights for the prediction and treatment of colon cancer.

## Materials and Methods

### Data Acquisition and Difference Analysis

From the Cancer Genome Atlas (TCGA^1^), the gene expression matrix and clinical information of 451 patients with colon cancer were obtained. The original data were normalized by robust multi-array average (RMA), and then the normalized value was log2 transformed. The normalized data were used for differential expression analysis. Differential expression gene analysis based on the limma function package of R language (version 3.5.2, the same below) was used to screen differentially expressed genes based on the absolute value of log 2FC > 1 and FDR ≤ 0.05.

### Construction and Model Construction of the ceRNA Network

We used the “GDCRNATools” package (Li et al., 2020) to integrate lncRNAs, miRNAs, and mRNAs differentially expressed in colon cancer. Referring to the starBase database, we associated the possible binding RNAs to construct the ceRNA network in colon cancer. According to the survival time of the patients, we screened out the RNAs in the ceRNA network that were significantly related to the prognosis of patients. Using lasso regression analysis, the risk score of each sample was calculated by the following formula:


$$\mathrm{Risk}\,\mathrm{score}=\sum_{i=1}^{n}{\mathrm{Coef}}_{i}*{\text{X}}_{i},$$

where Coef_*i*_ is the risk factor of each factor calculated by the lasso–Cox model, and x_*i*_ is the expression value of each factor, which in this study refers to the expression value of mRNA. Then, r-packet survival, survminer, and bilateral log-rank tests were used to determine the optimal cutoff value of the risk score. According to the cutoff value, patients were divided into low-risk and high-risk groups. We selected the best seven RNAs to construct the model.

### Model Validation and Nomogram Model Evaluation

We used the model in patients with colon cancer to test the overall survival rate of different groups based on the Kaplan–Meier method with R language survival package and survminer package and used the log-rank test to test the significance of survival rate difference between different groups. Time-dependent receiver operating characteristic (ROC) curve was drawn with R language survivalROC package (Heagerty et al., 2000). A multivariate Cox regression model was used to analyze whether risk score could predict the survival of patients with colon cancer independently of other factors. Nomogram is widely used to predict the prognosis of cancer. In order to predict the 1-, 3-, and 5-year survival probability of the patients, we established a nomogram based on all independent prognostic factors determined by multivariate Cox regression analysis, drew a nomogram calibration curve, and observed the relationship between nomogram prediction probability and actual incidence.

### Calculation of Immune Cell Infiltration Ratio and Tumor Purity

We used the software CIBERSORT (Newman et al., 2015) to calculate the relative proportion of 22 kinds of immune cells in each sample. According to the gene expression matrix, the CIBERSORT software uses the preset 547 barcode genes to represent the composition of immune-infiltrating cells by deconvolution algorithm. The sum of all estimated proportions of immune cell types in each sample is equal to 1. The R language estimate function package (Yoshihara et al., 2013) is used to calculate the tumor purity of each cancer sample. According to the results of CIBERSORT analysis, we analyzed and visualized the results of immune-infiltrating cells and drew the histogram of 22 kinds of immune cell infiltration in tumor tissues. The relationship between the expression levels of 22 kinds of immune cells in the samples was analyzed, and the correlation analysis chart of immune cells was drawn. The difference of the infiltration of immune cells in cancer tissues and normal tissues adjacent to cancer was compared, and the thermogram of immune cells and the violin diagram of difference analysis were drawn.

### Correlation Analysis of Immune Cells and Clinical Features

In order to explore the correlation between immune cells and the clinical stage, the data of immune cell expression and clinical stage were combined to analyze the relative expression of 22 kinds of immune cells in the T1–T4 stages. The expression data of the 22 kinds of immune cells were combined with survival data, and immune cell survival analysis (KM) was drawn, respectively.

### Construction and Validation of the Immune Cell Model

Firstly, the immune cell types that are significantly related to the prognosis of patients are preliminarily screened out through single-factor Cox regression analysis. Then, lasso regression analysis was used to remove the overfitting of the model, and the risk score of each sample was calculated by the following formula:


$$\mathrm{Risk}\,\mathrm{score}=\sum_{i=1}^{n}{\mathrm{Coef}}_{i}*{\text{X}}_{i},$$

where Coef_*i*_ is the risk factor of each factor calculated by the lasso–Cox model, and X_*i*_ is the expression value of each factor, which in this study refers to the relative expression of immune cells. Then, r-packet survival, survminer, and bilateral log-rank tests were used to determine the optimal cutoff value of the risk score. According to the cutoff value, patients were divided into low-risk and high-risk groups, and then the optimal immune cell was selected for model construction. Similarly, we validated the model using a data set containing the prognosis data of colon cancer patients, estimated the overall survival rate of different groups based on the Kaplan–Meier method using the R language survival package and survminer package, and used log-rank to test the significance of the difference in survival rate between different groups. The time-dependent ROC curve was drawn with R language survivalROC package (Heagerty et al., 2000). A multivariate Cox regression model was used to analyze whether risk score could predict the survival of patients with colon cancer independently of other factors, and finally, the most relevant immune cell types were selected. As in the previous steps, we used the nomogram model to predict 1-, 3-, and 5-year survival rates. Based on all independent prognostic factors determined by multivariate Cox regression analysis, a nomogram was established with the RMS package of the R language, and the nomogram calibration curve was drawn to observe the relationship between nomogram prediction probability and actual incidence.

### Risk Correlation Analysis and Co-expression Analysis of Immune Cells

The differential expression of immune cells screened by the model in the high- and low-risk groups (divided according to the risk score related to prognosis in the previous step) was compared and analyzed, and the risk heatmap of immune cells was drawn. The prognostic-related molecules and cells selected from the previous two models were summarized, and the correlation between molecules and molecules, and between molecules and cells, was analyzed, and the results were visualized. The co-expression analysis of the related molecules and immune cells was carried out, and the correlation coefficient and *P*-value were calculated and plotted.

### Enrichment Analysis and Protein–Protein Interaction Analysis of Prognostic Targets

The selected prognostic-related genes (lncRNA and miRNA in ceRNA are replaced by downstream target genes) and the marker genes of prognosis-related immune cells (CD16, CD32, TNF-α, MCP-1, CD86, CD64, CD80, CD8, CD205, Sirpa, CD11b, CD11, MHC II, CD45R, CD11c, TLR7, TLR9, IRF7, IRF8 and bdca2, CD1a, CD45, CD103-CD11b, CD1b, and CD1c) were analyzed. Cluster profiler R package was used for enrichment analysis, and online analysis tools of STRING^2^ and Metascape^3^ were used for protein interaction analysis.

### Clinical Sample Verification

Based on data from the HPA (The Human Protein Atlas) database, the expression levels of prognostic related immune cells in tumor tissues and in normal groups of patients with I and III stages of colon cancer were detected by immunohistochemistry.

## Results

### Construction and Model Construction of the Human Colon Cancer Cell Line CeRNA

**FIGURE 1:**
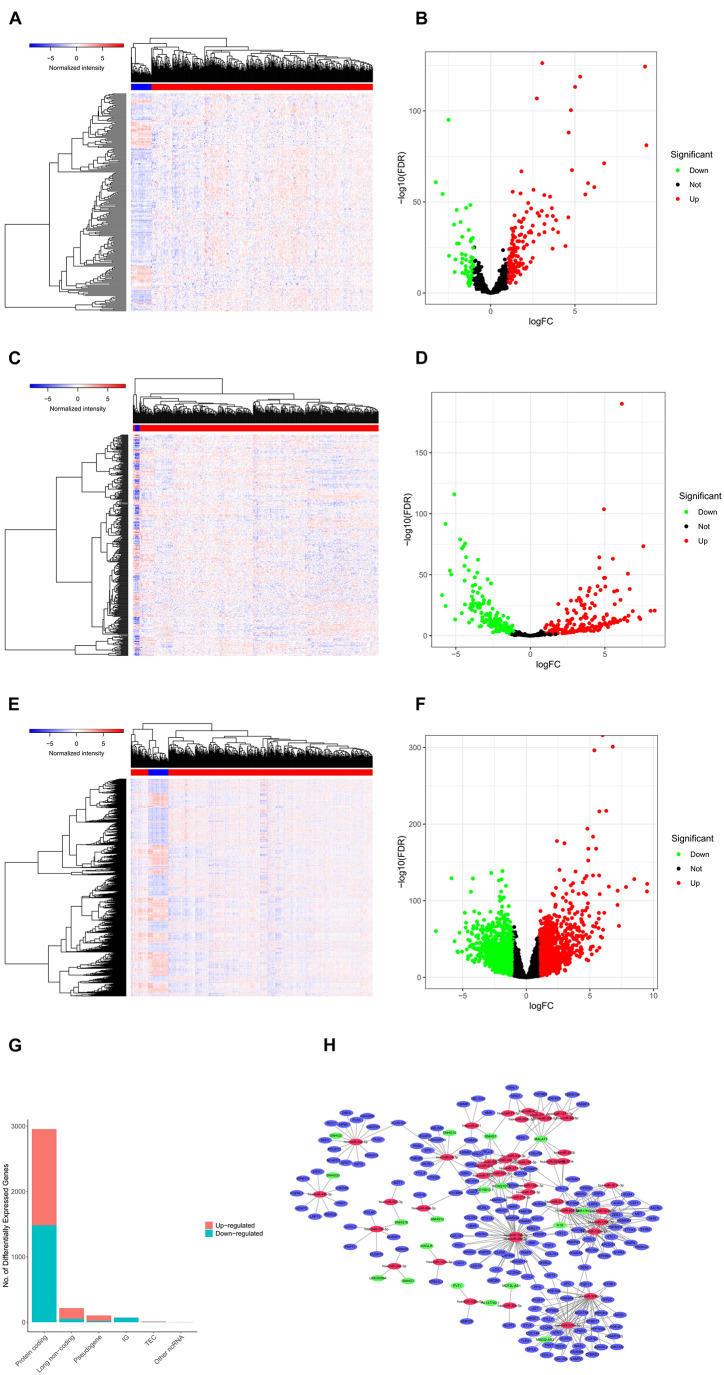
**(A)** Heatmap of differentially expressed lncRNAs in colon cancer. **(B)** Volcano map of differentially expressed lncRNAs in colon cancer. **(C,D)** Differentially expressed in colon cancer heatmap of miRNA. **(E,F)** Heatmap of differentially expressed mRNA in colon cancer. **(G)** Statistics of the upregulation of differentially expressed RNA. **(H)** Differential ceRNA regulatory network in colon cancer.

**FIGURE 2:**
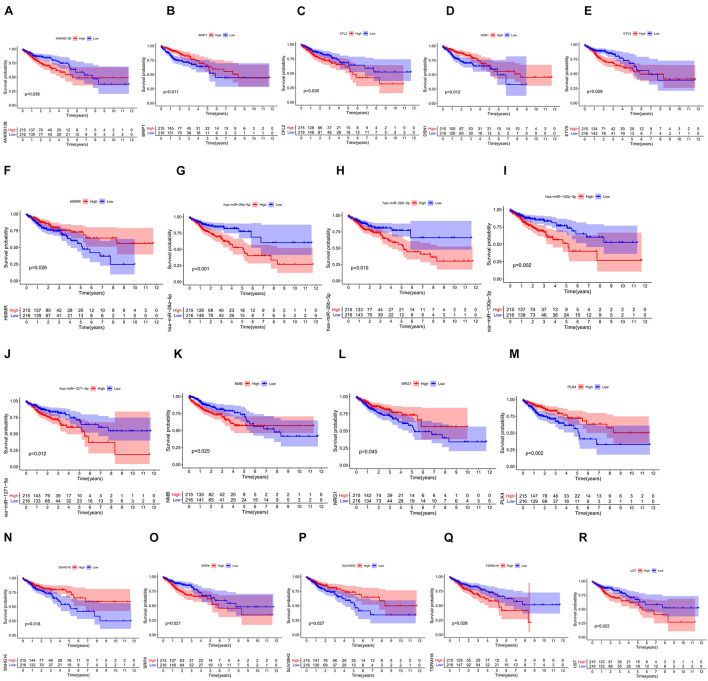
**(A–R)** Survival curves of 18 RNAs that are significantly related to the prognosis of colon cancer patients.

**FIGURE 3:**
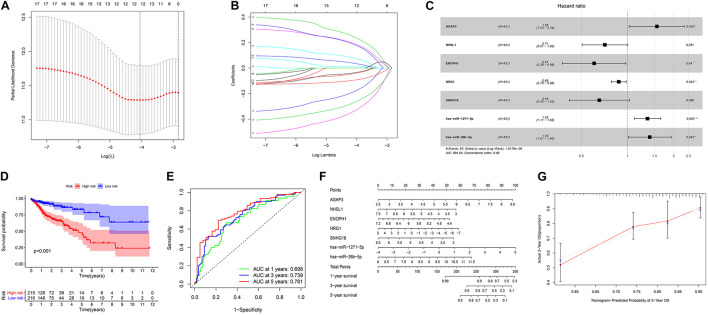
**(A)** Lasso regression analysis to screen prognostic-related genes. **(B)** Calculating the risk score of each patient and establishing the risk model. **(C)** Cox regression analysis of seven RNAs. **(D)** Comparison of prognostic differences between the high- and low-risk groups divided by the model. **(E)** Evaluation efficacy of the risk model at 1, 3, and 5 years. **(F)** Visual display of the nomogram patient prognosis evaluation model. **(G)** A 3-year evaluation efficacy of the nomogram model.

We screened 216 differentially expressed lncRNAs, 257 differentially expressed miRNAs, and 2,955 differentially expressed mRNAs in colon cancer by limma package and drew the corresponding heatmaps and volcanic maps (Figures 1A–G). Through the GDCRNATools package and reference to the starBase database, we linked the RNA that may be combined to construct a colon cancer ceRNA network, which includes 18 lncRNAs, 41 miRNAs, and 168 mRNA (Figure 1H). Combined with the survival time of patients with colon cancer, we screened out 18 RNAs significantly related to the prognosis of patients with colon cancer (Figures 2A–R). Through lasso regression analysis, we selected seven optimal RNA construction models, namely, AGAP3, NHSL1, ENOPH1, NRG1, SNHG16, hsa-mir-1271-5p, and hsa-mir-26b-5p, to predict the prognosis of patients with colon cancer (Figures 3A–C). The high expression of AGAP3, hsa-mir-1271-5p, and hsa-mir-26b-5p was the unfavorable factor of prognosis, and the high expression of ENOPH1 and NRG1 was the favorable factor of prognosis.

### Model Validation and Nomogram Model Evaluation

We used the model to divide colon cancer patients into high-risk and low-risk groups. Survival analysis showed that the survival rate of the high-risk group was significantly lower than that of the low-risk group (Figure 3D). Through the ROC curve analysis, we can see that the AUC values of the 3- and 5-year survival ROC curves of this model are greater than 0.7, indicating that the accuracy of this model is very high (Figure 3E). Then, the nomogram model was constructed according to the risk factors screened by the model, and the model was visualized as a nomogram to evaluate the correlation between the seven target molecules screened by the prognostic model and the prognosis of patients (Figures 3F,G).

### Infiltration of Immune Cells

**FIGURE 4:**
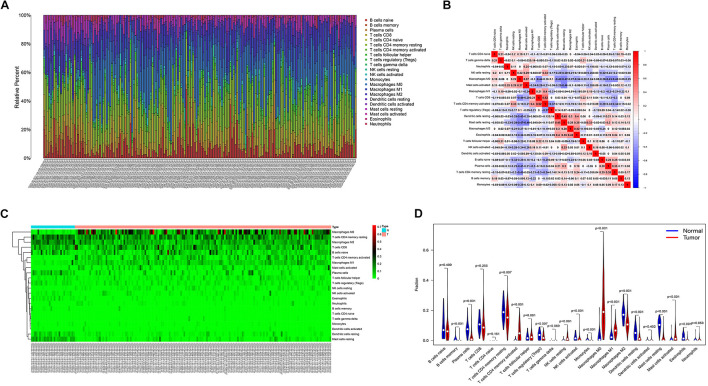
**(A)** Histogram of the infiltration ratio of the 22 immune cells in tumor tissue. **(B)** Correlation analysis of the respective expression levels of the 22 immune cells in the tumor sample. **(C)** Heatmap of the difference of immune cell infiltration between cancer tissue and normal tissue adjacent to cancer. **(D)** Violin image of the difference of immune cell infiltration difference between cancer tissue and normal tissue adjacent to cancer.

The proportion of immune cell infiltration in tumor tissues was visualized and a histogram was drawn (Figure 4A). The relationship between the expression levels of the 22 kinds of immune cells in the tumor samples was analyzed, and the correlation analysis chart of immune cells was drawn (Figure 4B). Differences in the infiltration of immune cells in cancer tissues and normal tissues adjacent to cancer were compared. The thermogram of immune cells and the violin diagram of difference analysis were drawn (Figures 4C,D).

### Correlation Analysis Between Immune Cells and Clinical Features

**FIGURE 5:**
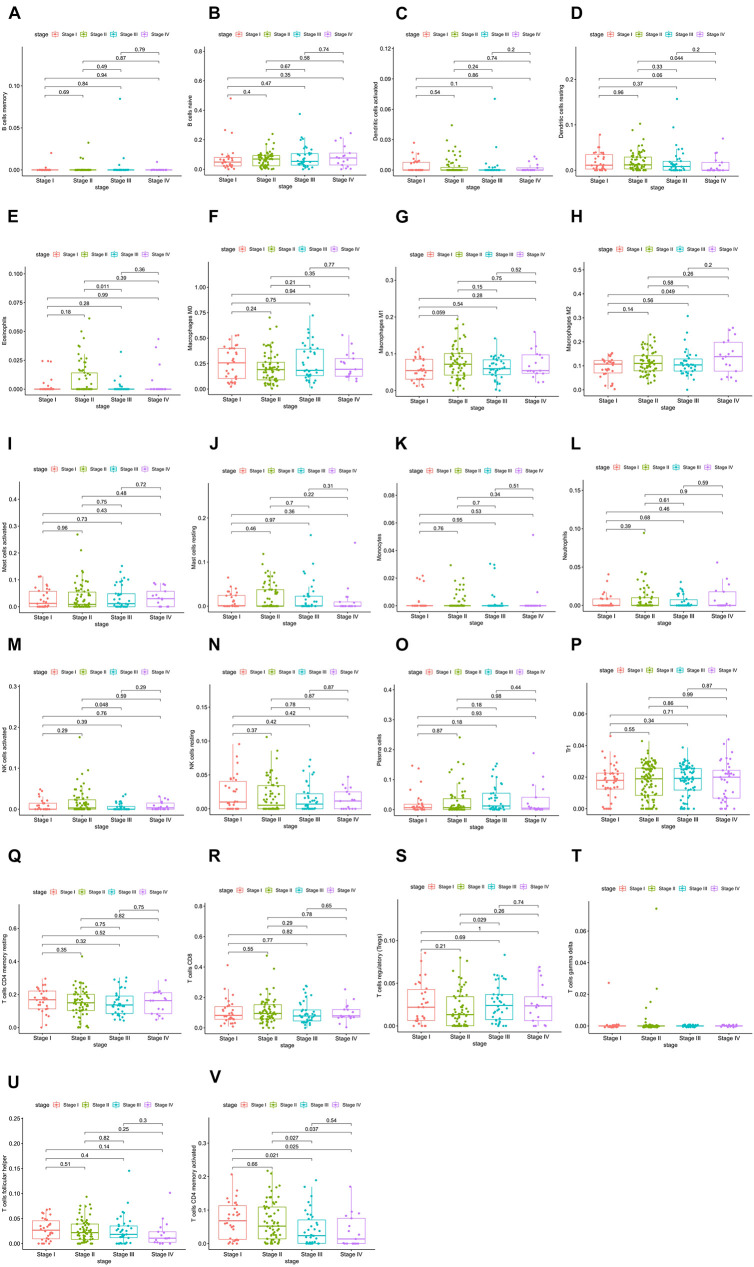
**(A–V)** Relative expression of the 22 immune cells in the T1–T4 stages of patients.

**FIGURE 6:**
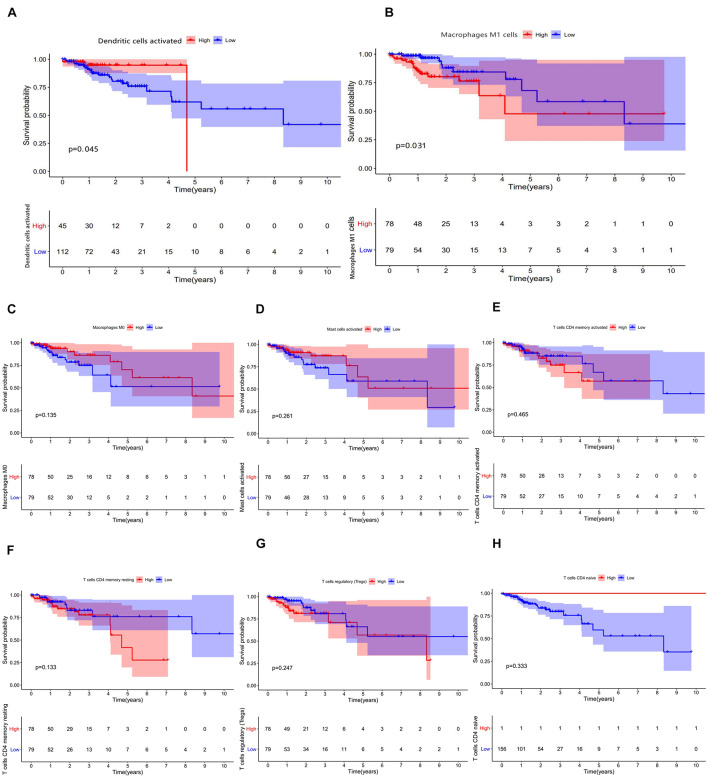
**(A–H)** Results of prognostic difference analysis of patients with high and low expression of immune cells.

The relative expression of the 22 kinds of immune cells in the T1–T4 stages of patients is shown in Figures 5A–V. We analyzed the differences in the expression of the 22 kinds of immune cells and the survival effect of patients. We found that patients with a high expression of macrophage M1 cells had a better prognosis than patients with a low expression of macrophage M1. The prognosis of patients with a high expression of dendritic cells of activated status was better than that of patients with low expression (Figures 6A–H).

### Construction and Validation of the Immune Cell Model

**FIGURE 7:**
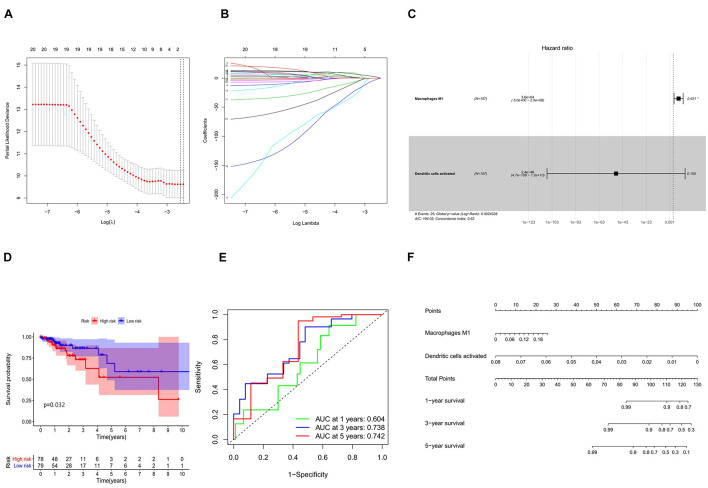
**(A)** Lasso regression analysis to screen immune cells related to prognosis. **(B)** Calculating the risk score of each patient and establishing the risk model. **(C)** Cox regression analysis of two prognostic-related immune cells. **(D)** High and low risk of immune cell model comparison of the prognosis of patients in the group. **(E)** The evaluation efficiency of the risk model at 1, 3, and 5 years. **(F)** The visual display of the nomogram patient prognosis evaluation model.

In combination with the survival time of patients with colon cancer, lasso and Cox regression analyses were used to further determine that macrophages M1 and activated dendritic cells are the most significantly correlated with the prognosis of patients with colon cancer, and a prognosis model was established based on these two immune cells (Figures 7A–D). The results of ROC analysis showed that the AUC values of the ROC curves for the 3- and 5-year survival rates were greater than 0.7, indicating that the accuracy of the model was very high (Figure 7E). The nomogram model validation results are similar (Figure 7F).

### Immunocyte Risk Thermography, Immunocyte Correlation Analysis, and Co-expression Analysis

**FIGURE 8:**
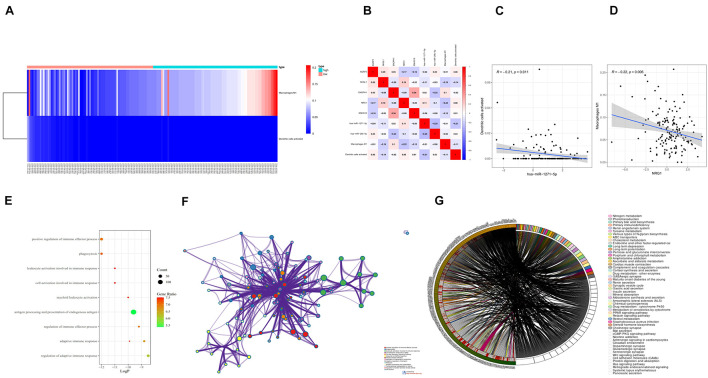
**(A)** Heatmap of the differential expression of two immune cells in the high- and low-risk groups. **(B)** Prognostic-related molecules and cell correlation analysis results. **(C)** Dendritic cells activated and hsa-miR-1271-5p molecules. Correlation analysis results of **(D)** macrophage M1 cells and NRG1 molecules. **(E)** Bubble chart of GO enrichment analysis of prognostic-related genes. **(F)** Network chart of GO enrichment analysis of prognostic-related genes. **(G)** Circle diagram of KEGG enrichment analysis of prognostic-related genes.

The differential expression of the two immune cells in the high- and low-risk groups is shown in the immunocyte risk heatmap (Figure 8A). The correlation analysis of prognostic-related molecules and cells included in the two prognostic models showed that ENOPH1 had the highest correlation with the *snhg16* gene (0.34). The specific correlation analysis results are shown in Figure 8B. Correlation analysis showed that the highest correlation was found between activated dendritic cells and hsa-mir-1271-5p. The co-expression analysis showed that the correlation coefficient was –0.21, *P* = 0.011 (Figure 8C). The strongest correlation was found between macrophage M1 cells and NRG1, with a correlation coefficient of –0.22, *P* = 0.006 (Figure 8D).

### Enrichment Analysis and Protein Interaction Analysis of Prognostic Targets

**FIGURE 9:**
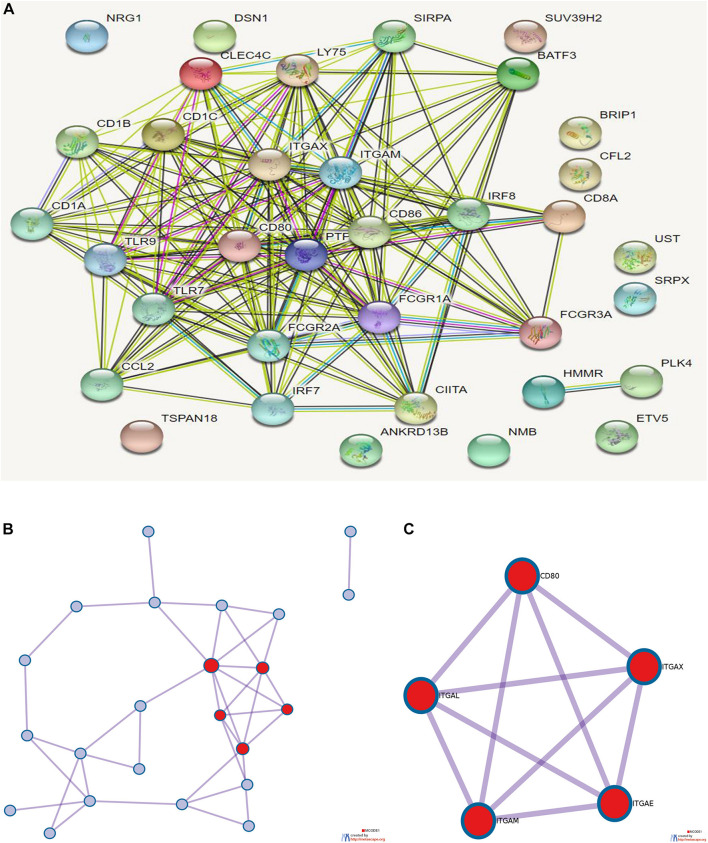
**(A)** Protein interaction analysis results of prognostic-related genes. **(B)** Topological network of protein interaction analysis results. **(C)** Core genes screened by protein interaction analysis. qPCR detection of the seven core RNAs in phases I and III and differential expression in tissues of patients with colon cancer and normal tissues.

Eighteen prognostic-related genes and marker genes of prognosis-related immune cells were analyzed. Enrichment analysis showed that the prognosis-related genes were mainly related to biological processes such as positive regulation of immune effector process, phagocytosis, leucocyte activation involved in immune response, cell activation involved in immune response, and myeloid leukocyte activation (Figures 8E,F). Kyoto Encyclopedia of Genes and Genomes (KEGG) enrichment analysis showed that prognosis-related genes were mainly enriched in the related signaling pathways such as hematopoietic cell lineage, tuberculosis, cell adhesion molecules (CAMs), and toll-like receptor signaling pathway (Figure 8G). The protein–protein interaction analysis results are shown in Figures 9A–C. The results show that *CD80*, *ITGAL*, *ITGAX*, *ITGAM*, *ITGAE*, and other genes are the core genes and interact most closely with other targets.

### Clinical Sample Verification

**FIGURE 10:**
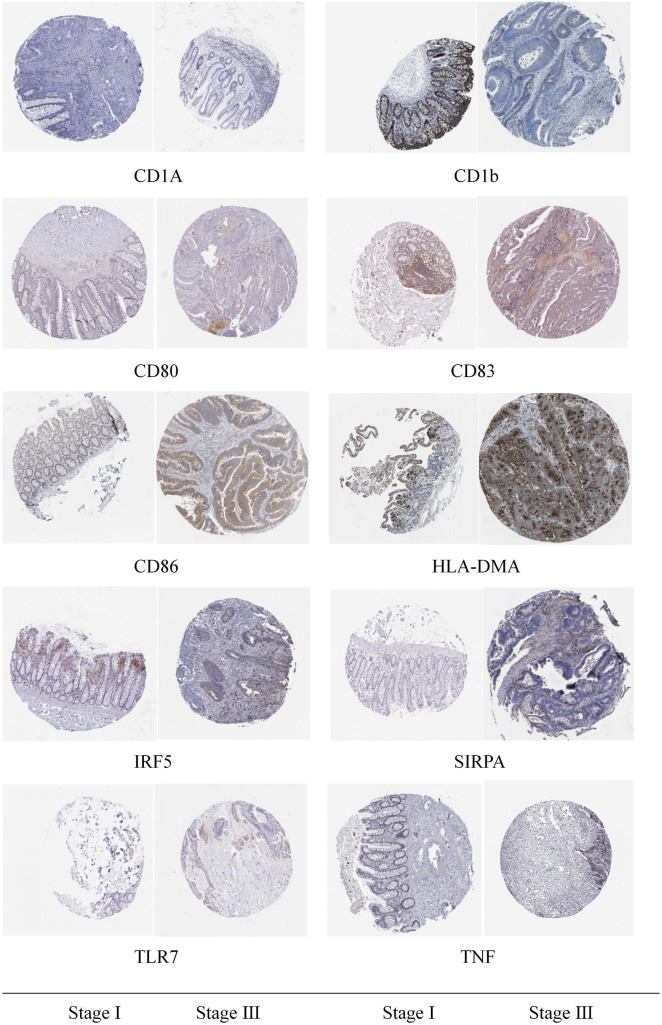
Immunohistochemical detection results of the marker genes of M1 macrophages and dendritic cells.

Through immunohistochemical detection of markers of M1 macrophages and dendritic cells, we found that compared with patients with stage I colon cancer, markers of M1 macrophages are expressed in higher levels in the tumor tissues of patients with stage III colon cancer. Compared with patients with stage I colon cancer, the markers of dendritic cells are lower in the tumor tissues of patients with stage III colon cancer (Figure 10).

## Discussion

Mortality due to colon cancer is related to the stage of the disease. The 5-year survival rate of patients with stage I can reach more than 90% (Heagerty et al., 2000; Yoshihara et al., 2013; Newman et al., 2015), but for patients with metastasis, the 5-year survival rate is only 10%–20%. Therefore, the mortality rate of colon cancer is the second highest in the world (Engelhardt et al., 2017; Peng et al., 2019). Surgical resection and chemotherapy are the routine treatment for patients with colon cancer, but in one study, patients with colon cancer after conventional treatment still showed a high recurrence rate and mortality (Weinberg et al., 2017). Therefore, it is very important to predict the prognosis of patients with colon cancer and to give individualized treatment. In this study, we identified seven kinds of mRNAs as molecular markers of prognosis in patients with colon cancer and further screened out two kinds of immune cells related to prognosis, which proved that the combination of seven RNAs and two kinds of immune cells can reliably evaluate the prognosis of patients with colon cancer.

Snhg16 has been proven to affect the viability of colon cancer cells. Silencing snhg16 can significantly increase apoptosis and reduce the migration of colon cancer cells, and snhg16 also participates in the lipid metabolism of colon cancer cells (Christensen et al., 2016). The expression of ENOPH1 was found to be increased in gliomas, while inhibition of ENOPH1 significantly reduced cell proliferation and cell migration (Su et al., 2018). NRG1 is a ligand of HER3 and HER4 receptors, which is secreted by pancreatic tumor cells. The increase of NRG1 will interfere with the effect of chemotherapy on pancreatic ductal adenocarcinoma. Therefore, anti-NRG1 may represent a new method for targeting the pancreatic matrix and cancer cells (Ogier et al., 2018). Two miRNAs, mir-1271-5p and mir-26b-5p, were found to play an important role in cancer (Khosla et al., 2019). Zhou et al. (Zhou et al., 2020) found that mir-26b-5p could inhibit the proliferation, migration, and invasion of papillary thyroid cancer in a β-catenin-dependent manner. MicroRNA-26b-5p was also found to have the ability to enhance T-cell responses by targeting Pim-2 in hepatocellular carcinoma (Lin et al., 2017; Han et al., 2019). The study suggested that lncRNA RP11-619L19.2 could promote colon cancer development by regulating the miR-1271-5p/CD164 axis (Zhang et al., 2020). Fan et al. (2020) found that upregulation of lncRNA ZFAS1 could promote lung adenocarcinoma progression by sponging miR-1271-5p and upregulating FRS2. Chen et al. (2019) found that miR-1271-5p could inhibit cell proliferation and induce apoptosis in acute myeloid leukemia by targeting ZIC2.

As the main antigen-presenting cells, activated dendritic cells have been confirmed by many studies to be related to the occurrence and development of malignant tumors (Veglia and Gabrilovich, 2017). In our study, we found that the expression of activated dendritic cells in the high-risk group was low, that is, the low expression of activated dendritic cells was associated with poor prognosis. Through further analysis, the relationship between the expression of activated dendritic cells and hsa-miR-1271-5p was found. Macrophages are the most abundant immune cells in the tumor microenvironment, which can secrete a variety of cytokines. At the early stage of tumor occurrence, it can recognize and remove tumor cells. However, with the development of tumors, it plays a key role in tumor growth, invasion, and metastasis (Cassetta and Pollard, 2018; Pathria et al., 2019). Macrophages play a “double-edged sword” role in the occurrence and development of tumors. Influenced by the cytokines in the tumor microenvironment, macrophages differentiate into different types, mainly divided into M1 type and M2 type (Orecchioni et al., 2019). In each stage of the tumor, M1 and M2 macrophages were present; the M1 type was the main type in the early stage, and the M2 type was the main type in the middle and late stages. It is generally believed that M1 TAM can release a variety of proinflammatory factors, immune-activating factors, and chemokines and play an anti-tumor role through acute proinflammatory reaction, immune activation reaction, and phagocytosis function (Travers et al., 2019; Liu et al., 2020). With the development of tumors, the M1 type gradually polarizes to the M2 type, and the increase of M2-type TAM also indicates a poor prognosis. However, our study found that macrophage M1 cells had the strongest correlation with NRG1 molecules, and macrophage M1 was highly expressed in the high-risk group, indicating that the relative proportion of increased macrophage M1 was associated with poor prognosis of patients. This may be due to the fact that our calculated expression of macrophage M1 cells is its relative expression in 22 kinds of immune cells. When its relative proportion increases, the proportion of other anti-tumor immune cells such as T cells is relatively reduced, which may be the cause of poor prognosis of patients.

There are several previous studies based on bioinformatics analysis that screen prognostic markers of colon cancer patients, but most of them only focus on the transcriptome level. However, our study focused on the effect of non-coding RNA and mRNA on the prognosis of colon cancer patients and constructed the lncRNA–miRNA–mRNA co-expression regulatory network. In our study, non-coding RNA is also included in the construction of the prognosis model, which enables our research to obtain a more scientific and reliable biological basis. In addition, we added research on the correlation between immune cells and the prognosis of patients for the first time and combined it with selected RNA molecules to comprehensively evaluate the prognosis of patients, which provided a new idea for exploring the progress of colon cancer disease and the prognosis of patients from the cellular level. Finally, we used a large number of tissue samples from patients with different clinical stages to verify the differential expression of seven RNA molecules. The experimental results were basically consistent with the prediction results of our model, which confirmed the reliability of the research conclusion and the effectiveness of the seven RNA molecules as prognostic biomarkers.

## Conclusion

In general, our study constructed the differentially expressed ceRNA network of colon cancer patients, screened out seven RNA molecules significantly related to the survival of patients, and successfully constructed the prognosis evaluation model of patients. We further screened two kinds of immune cells related to the prognosis of patients and constructed the corresponding prognosis evaluation model, combined with seven RNA molecules to predict the prognosis of patients with colon cancer more accurately and comprehensively. The results showed that the expression of seven RNA markers in colon cancer patients with different prognoses was basically consistent with the predicted results of the model, which further demonstrated that these biomarkers we screened can effectively evaluate the prognosis of patients with colon cancer.

## Data Availability Statement

The datasets presented in this study can be found in online repositories. The names of the repository/repositories and accession number(s) can be found in the article/supplementary material.

## Author Contributions

JX contributed to the research design and manuscript writing. TL contributed to the data analysis, and model validation and modification of the manuscript. SD contributed to the manuscript revision and language modification. SH contributed to the reference search, manuscript review, and manuscript revision. KD guided the manuscript writing, assigned work tasks, and provided funding support.

## Conflict of Interest

The authors declare that the research was conducted in the absence of any commercial or financial relationships that could be construed as a potential conflict of interest.

## Publisher’s Note

All claims expressed in this article are solely those of the authors and do not necessarily represent those of their affiliated organizations, or those of the publisher, the editors and the reviewers. Any product that may be evaluated in this article, or claim that may be made by its manufacturer, is not guaranteed or endorsed by the publisher.

## Footnotes

1
https://tcga-data.nci.nih.gov/tcga/

2
https://www.string-db.org/

3
http://metascape.org/

## References

[B1] American Cancer Society Cancer Facts (2019). Available online at: https://www.cancer.org/content/dam/cancer-org/research/cancer-facts-and-statistics/annual-cancer-facts-and-figures/2019/cancer-facts-and-figures-2019.pdf. (accessed January. 4, 2020)

[B2] Cassetta L. Pollard J. W. (2018). Targeting macrophages: therapeutic approaches in cancer. *Nat. Rev. Drug. Dis.* 17 887–904. 10.1038/nrd.2018.169 30361552

[B3] Chen X. Yang S. Zeng J. Chen M. (2019). miR-1271-5p inhibits cell proliferation and induces apoptosis in acutemyeloid leukemia by targeting ZIC2[J]. *Mol. Med. Rep.* 19 508–514. 30483794 10.3892/mmr.2018.9680 PMC6297795

[B4] Christensen L. L. True K. Hamilton M. P. Nielsen M. M. Damas N. D. Damgaard C. K. (2016). SNHG16 is regulated by the Wnt pathway in colorectal cancer and affects genes involved in lipid metabolism. *Mol. Oncol.* 10 1266–1282. 10.1016/j.molonc.2016.06.003 27396952 PMC5423192

[B5] Engelhardt E. G. Révész D. Tamminga H. J. Punt C. J. Koopman M. Onwuteaka-Philipsen B. D. (2017). Clinical usefulness of tools to support decision-making for palliative treatment of metastatic colorectal cancer: a systematic review. *Clin. Colorectal. Cancer* 17 e1–e12. 10.1016/j.clcc.2017.06.007 28734786

[B6] Fan G. Jiao J. Shen F. Chu F. (2020). Upregulation of lncRNA ZFAS1 promotes lung adenocarcinoma progression by sponging miR-1271-5p and upregulating FRS2. *Thor. Cancer* 11 2178–2187. 10.1111/1759-7714.13525 32515146 PMC7396366

[B7] FANTOM Consortium, Okazaki Y. Furuno M. Kasukawa T. Adachi J. Bono K. (2002). “Analysis of the mouse transcriptome based on functional annotation of 60,770 full-length cDNAs”. *Nature* 420 563–573. 10.1038/nature01266 12466851

[B8] Guttman M. Rinn J. L. (2012). Modular regulatory principles of large non-coding RNAs. *Nature* 482 339–346. 10.1038/nature10887 22337053 PMC4197003

[B9] Han W. Li N. Liu J. Sun Y. Yang X. Wang Y. (2019). MicroRNA-26b-5p enhances T cell responses by targeting PIM-2 in hepatocellular carcinoma. *Cell. Sign.* 59 182–190. 10.1016/j.cellsig.2018.11.011 30593845

[B10] Heagerty P. J. Lumley T. Pepe M. S. (2000). Time-dependent ROC curves for censored survival data and a diagnostic marker. *Biometrics* 56 337–344. 10.1111/j.0006-341x.2000.00337.x 10877287

[B11] Huxley R. R. Ansary-Moghaddam A. Clifton P. Czernichow S. Parr C. L. Woodward M. (2009). The impact of dietary and lifestyle risk factors on risk of colorectal cancer: a quantitative overview of the epidemiological evidence. *Int. J. Cancer* 125 171–180. 10.1002/ijc.24343 19350627

[B12] Johns L. E. Houlston R. S. (2001). A systematic review and meta-analysis of familial colorectal cancer risk. *Am. J. Gastroenter.* 96 2992–3003. 10.1111/j.1572-0241.2001.04677.x 11693338

[B13] Kapranov P. Cheng J. Dike S. Nix D. A. Duttagupta R. Willingham A. T. (2007). RNA maps reveal new RNA classes and a possible function for pervasive transcription. *Science* 316 1484–1488. 10.1126/science.1138341 17510325

[B14] Khosla R. Hemati H. Rastogi A. Ramakrishna G. Sarin S. K. Trehanpati N. (2019). miR-26b-5p helps in EpCAM+cancer stem cells maintenance via HSC71/HSPA8 and augments malignant features in HCC. *Liver Int.* 39 1692–1703. 10.1111/liv.14188 31276277

[B15] Li R. Qu H. Wang S. Wei J. Zhang L. Ma R. (2020). *GDCRNATools: GDCRNATools: An R/Bioconductor Package for Integrative Analysis of lncRNA, mRNA, and miRNA Data in GDC. R Package Version 1.8.0.* 10.1093/bioinformatics/bty124 29509844

[B16] Lin M. F. Yang Y. F. Peng Z. P. Zhang M. F. Liang J. Y. Chen W. (2017). FOXK2, regulted by miR-1271-5p, promotes cell growth and indicates unfavorable prognosis in hepatocellular carcinoma. *Int. J. Biochem. Cell Biol.* 88 155–161. 10.1016/j.biocel.2017.05.019 28506857

[B17] Liu Z. Xie Y. Xiong Y. Liu S. Qiu C. Zhu Z. (2020). TLR 7/8 agonist reverses oxaliplatin resistance in colorectal cancer via directing the myeloid-derived suppressor cells to tumoricidal M1-macrophages. *Cancer Lett.* 469 173–185. 10.1016/j.canlet.2019.10.020 31629935

[B18] Meyerhardt J. A. Catalano P. J. Haller D. G. Mayer R. J. Macdonald J. S. Benson A. B. (2003). Impact of diabetes mellitus on outcomes in patients with colon cancer. *J. Clin. Oncol.* 21 433–440. 10.1200/JCO.2003.07.125 12560431

[B19] Newman A. M. Liu C. L. Green M. R. Gentles A. J. Feng W. Xu Y. (2015). Robust enumeration of cell subsets from tissue expression profiles. *Nat. Methods.* 12 453–457. 10.1038/nmeth.3337 25822800 PMC4739640

[B20] O’eilly M. S. Boehm T. Shing Y. Fukai N. Vasios G. Lane W. S. (1997). Endostatin: an endogenous inhibitor of angiogenesis and tumor growth. *Cell* 88 277–285. 10.1016/S0092-8674(00)81848-6 9008168

[B21] Ogier C. Colombo P. E. Bousquet C. Canterel-Thouennon L. Sicard P. Garambois V. (2018). Targeting the NRG1/HER3 pathway in tumor cells and cancer-associated fibroblasts with an anti-neuregulin 1 antibody inhibits tumor growth in pre-clinical models of pancreatic cancer. *Cancer Lett.* 432 227–236. 10.1016/j.canlet.2018.06.023 29935372

[B22] Orecchioni M. Ghosheh Y. Pramod A. B. Ley K. (2019). Macrophage polarization: different gene signatures in M1(LPS+) vs. classically and M2(LPS-) vs. alternatively activated macrophages. *Front. Immunol.* 10:1084. 10.3389/fimmu.2019.01084 PMC6543837 31178859

[B23] Pathria P. Louis T. L. Varner J. A. (2019). Targeting tumor-associated macrophages in cancer. *Trends Immunol.* 40 310–327. 30890304 10.1016/j.it.2019.02.003

[B24] Peng Z. Ruidong L. Hua X. Liu W. Zeng X. Xie G. (2019). BRD4 inhibitor AZD5153 suppresses the proliferation of colorectal cancer cells and sensitizes the anticancer effect of parp inhibitor. *Int. J. Biol. Sci.* 15 1942–1954. 10.7150/ijbs.34162 31523195 PMC6743290

[B25] Qi X. Zhang D.-H. Wu N. Xiao J.-H. Wang X. Ma W. (2015). ceRNA in cancer: possible functions and clinical implications. *J. Med. Genet.* 52 710–718. 10.1136/jmedgenet-2015-103334 26358722

[B26] Siegel R. L. Miller K. D. Jemal A. (2017). Cancer statistics, 2017. *CA Cancer J. Clin.* 67 7–30. 10.3322/caac.21387 28055103

[B27] Su L. Yang K. Li S. Liu C. Han J. Zhang Y. (2018). Enolase-phosphatase 1 as a novel potential malignant glioma indicator promotes cell proliferation and migration. *Oncol. Rep.* 40 2233–2241. 10.3892/or.2018.6592 30066900

[B28] Sung J. J. Lau J. Y. Goh K. L. Leung W. K. (2005). Increasing incidence of colorectal cancer in Asia: implications for screening. *Lancet Oncol.* 6 871–876. 10.1016/s1470-2045(05)70422-8 16257795

[B29] Tay Y. Rinn J. Pandolfi P. P. (2014). The multilayered complexity of ceRNA crosstalk and competition. *Nature* 505 344–352. 10.1038/nature12986 24429633 PMC4113481

[B30] Terzić J. Grivennikov S. Karin E. Karin M. (2010). Inflammation and colon cancer. *Gastroenterology* 138 2101–2114.e5. 10.1053/j.gastro.2010.01.058 20420949

[B31] Travers M. Brown S. M. Dunworth M. Holbert C. E. Wiehagen K. R. Bachman K. E. (2019). DFMO and 5-azacytidine increase M1 macrophages in the tumor microenvironment of murine ovarian cancer. *Cancer Res.* 79 3445–3454. 10.1158/0008-5472.can-18-4018 31088836 PMC6606334

[B32] Vatandoust S. Price T. J. Karapetis C. S. (2015). Colorectal cancer: metastases to a single organ. *World J. Gastroenterol.* 21 11767–11776. 10.3748/wjg.v21.i41.11767 26557001 PMC4631975

[B33] Veglia F. Gabrilovich D. I. (2017). Dendritic cells in cancer: the role revisited. *Curr. Opin. Immunol.* 45 43–51. 10.1016/j.coi.2017.01.002 28192720 PMC5449252

[B34] Watson A. J. M. Collins P. D. (2011). Colon cancer: a civilization disorder. *Digest. Dis.* 29 222–228. 10.1159/000323926 21734388

[B35] Weinberg B. A. Marshall J. L. Salem M. E. (2017). The Growing Challenge of young adults with colorectal cancer. *Oncology (Williston Park)* 31 381–389. 28516436

[B36] Wu Q. Meng W. Y. Jie Y. Zhao H. (2018). LncRNA MALAT1 induces colon cancer development by regulating miR-129-5p/HMGB1 axis. *J. Cell Physiol.* 233 6750–6757. 10.1002/jcp.26383 29226325 PMC13482480

[B37] Xu J. Li Y. Lu J. Pan T. Ding N. Wang Z. (2015). “The mRNA related ceRNA-ceRNA landscape and significance across 20 major cancer types”. *Nucleic Acids Res.* 43 8169–8182. 10.1093/nar/gkv853 26304537 PMC4787795

[B38] Yoshihara K. Shahmoradgoli M. Martínez E. Vegesna R. Kim H. Torres-Garcia W. (2013). Inferring tumour purity and stromal and immune cell admixture from expression data. *Nat. Commun.* 4:2612. 10.1038/ncomms3612 24113773 PMC3826632

[B39] Zhang X. W. Li S. L. Zhang D. Sun X. L. Zhai H. J. (2020). RP11-619L19.2 promotes colon cancer development by regulating the miR-1271-5p/CD164 axis.[J]. *Oncol. Rep.* 44 2419–2428. 10.3892/or.2020.7794 33125110 PMC7610312

[B40] Zhou A. Pan H. Sun D. Xu H. Zhang C. Chen X. (2020). miR-26b-5p inhibits the proliferation, migration and invasion of human papillary thyroid cancer in a β-catenin-dependent manner. *OncoTargets Ther.* 13 1593–1603. 10.2147/ott.s236319 32110056 PMC7041607

